# MCMs in Cancer: Prognostic Potential and Mechanisms

**DOI:** 10.1155/2020/3750294

**Published:** 2020-02-03

**Authors:** Si Yu, Guanqun Wang, Yue Shi, Haifeng Xu, Yongchang Zheng, Yang Chen

**Affiliations:** ^1^Department of Liver Surgery, Peking Union Medical College Hospital, Chinese Academy of Medical Sciences & Peking Union Medical College, Beijing 100730, China; ^2^School of Life Sciences, Tsinghua-Peking Joint Center for Life Sciences, Center for Synthetic and Systems Biology, Ministry of Education Key Laboratory of Bioinformatics, Tsinghua University, Beijing 100084, China; ^3^MOE Key Laboratory of Bioinformatics, Center for Synthetic and Systems Biology, Bioinformatics Division, BNRist, Department of Automation, Tsinghua University, Beijing 100084, China

## Abstract

Enabling replicative immortality and uncontrolled cell cycle are hallmarks of cancer cells. Minichromosome maintenance proteins (MCMs) exhibit helicase activity in replication initiation and play vital roles in controlling replication times within a cell cycle. Overexpressed MCMs are detected in various cancerous tissues and cancer cell lines. Previous studies have proposed MCMs as promising proliferation markers in cancers, while the prognostic values remain controversial and the underlying mechanisms remain unascertained. This review provides an overview of the significant findings regarding the cellular and tumorigenic functions of the MCM family. Besides, current evidence of the prognostic roles of MCMs is retrospectively reviewed. This work also offers insight into the mechanisms of MCMs prompting carcinogenesis and adverse prognosis, providing information for future research. Finally, MCMs in liver cancer are specifically discussed, and future perspectives are provided.

## 1. MCM Family and Cellular Functions

Minichromosome maintenance proteins (MCMs) were first identified in *Saccharomyces cerevisiae* as imperative factors in the maintenance of extrachromosomal DNA [1]. In eukaryotic cells, MCM2-7 form a hetero-hexameric AAA+ ATPase. A structural and biochemical study showed that in the complex, the central AAA+ domain had helicase activity while the N-terminal domain functioned as an organizing center in *trans*-acting replisome [2].

**Figure 1:**
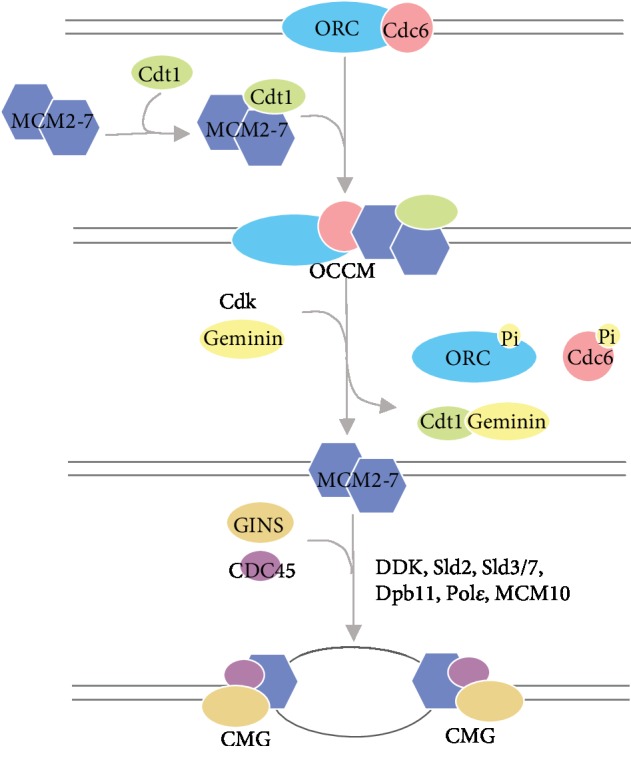
MCMs play essential roles in DNA replication initiation. During the G1 phase, ORC and Cdc6 bind to condensed chromatin in advance and recruit MCM2-7 double hexamer and Cdt1 to form OCCM intermediate. During the S phase, ORC and Cdc6 exit via Cdk-dependent phosphorylation and Cdt1 exits via geminin-dependent ubiquitination. The double hexamer separates and forms CMG complexes separately. GINS and CDC45 as well as other initiation factors interact with MCM2-7 hexamer and trigger DNA unwinding [3, 6, 7].

MCMs play essential roles as a helicase and organizing center in DNA replication initiation (Figure 1). During the late M to G1 phase, the origin recognition complex (ORC) and Cdc6 are initially recruited on the replication origin [3]. Afterward, randomly distributed double hexamers of MCM2-7 interact with Cdt1, the MCM loader, and the two subunits engage in the formation of ORC-Cdc6-Cdt1-Mcm2-7 (OCCM) intermediate, functioning as a component of the prereplication complex (pre-RC) [4]. In the S phase, ORC1 and CDC6 are phosphorylated by activated CDK kinase and detached from the origin. Cdt1 is degraded via geminin-mediated ubiquitination. CDK and other initiation factors (DDK, Sld2, Sld3/7, Dpb11, Pol*ε*, CDC45, and GINS) facilitate the activation of helicase activity in MCM2-7. The double hexamers separate and individually associate with CDC45 and GINS to form the CDC45-MCM-GINS (CMG) complex. CMG complex triggers the unwinding of DNA at the origin and serves as the platform for complete replisome assembly [5, 6].

Particularly, MCMs are functionally related to accurate DNA replication licensing. In a dividing cell, MCM2-7 are the only proteins present in all stages of the cell cycle [3], and they are strictly regulated at the steps of elongation and termination [8]. The switch between active/inactive states would ensure that DNA only replicates once at a time. Meanwhile, MCMs also provide flexibility, redundancy, and robustness for the genome. Only a fraction of the excess MCM2-7 proteins bound to chromatin are located in ORC binding site and activated in any S phase. The displaced MCM2-7 proteins are still functional in initiation [8], providing an intrinsically flexible replication strategy, which could be crucial in proliferation regulation and replication stress response.

In the MCM family, MCM subunits have distinct functions in chromosomal DNA replication (Table 1). During initiation, MCM2/4 interact with the MCM loader Cdt1 at NTD (N-terminus domain) to stabilize the coil structure [4]. MCM2/5 form a gate for the MCM2-7 loading [13]. In the OCCM intermediate, MCM3 interacts with ORC·Cdc6 at CTD and stimulates the ATPase activity [14]. In the CMG complex, MCM2/5 and MCM3/5 correspondingly interact with Cdc45 and GINS, and the association is stabilized by MCM10; MCM10 also helps activate the helicase activity of MCM2-7 complex and stimulates elongation [12, 15, 20, 21]. During elongation, MCM4/6/7 is directly involved in the translocation of CMG complex from the origin along the single-strand DNA in the MCM2-7COMPLEX, and MCM2 can be dephosphorylated by PTEN to restrict replication fork progression [1, 11]. MCM2/4/5/6/7 correlatedly shifted and formed a tight cluster [23]. In metazoans, MCM8 can function as a helicase, and MCM10 may coordinate DNA helicase and polymerization [18, 22]. During termination, MCM7 is involved in MCM2-7 degradation in a ubiquitylation-dependent manner [16]. Beyond the process of DNA replication, MCM2 has histone-handling activity, and the MCM8-9 is demonstrated to facilitate homologous recombination at DNA damage sites by promoting RAD51 recruitment [9, 18]. Besides, the trimeric MCM4/6/7 was demonstrated to be competent to unwind DNA in vitro [24].

## 2. MCMs and Cancer

### 2.1. MCMs in Cancer Development

Unlimited DNA replication is one of the characteristics of dysplasia and malignant cells [25]. Many DNA replication proteins have been proposed to be promising cancer biomarkers. MCM proteins are suggested to be closely related to cancer development as well. Overexpression of MCMs has been detected in various cancer tissues and carcinoma cell lines including lung squamous cell carcinoma [26], renal cell carcinoma [27], prostate cancer [28], breast cancer [29], gastrointestinal tract tumors [30–32], brain tumors [33, 34], and lymphomas [35, 36]. Studies have also shown the links between critical clinicopathological characteristics and upregulated MCMs in some cancers [37, 38]. Besides, expression levels of MCM proteins are reported to be associated with different malignant differentiated degrees, clinical stages, and tumor sizes [26, 39].

Since MCMs are indispensable for DNA replication licensing and significantly downregulated when the cell exits from the cell cycle into quiescence (G0), differentiation, or senescence [40–42], high levels of MCMs are proposed as potential proliferation markers. MCM-knockdown, expression microarray, and immunohistochemical analysis are used to identify MCMs as proliferation markers in gastric tumors [43], oral squamous cell carcinoma [44], ovarian cancer [45], and salivary gland tumors [46]. MCM3, MCM4, and MCM7 have advantages over traditional cell-cycle markers like Ki67 and proliferating cell nuclear antigen (PCNA) because they have higher sensitivity and are less likely to be influenced by external factors like inflammatory factors [44, 47]. The presence of MCMs in all cell stage may account for the superiority, considering that expression of Ki67 and PCNA can only be observed in certain stages of replication and can be easily disturbed [48, 49].

In addition to overproliferation, upregulated MCMs are also related to cancer metastasis. Anchorage-independent cell growth, cell migration, and invasion were improved in medulloblastoma with exogenous overexpressed MCM2, MCM3, and MCM7 [50]. At the same time, MCM8 has also been reported to be involved in chromosome rearrangement [18]. Numerous rearrangements are one of the features of the aggressive cancer genome. The decreased volume of xenografted tumor in MCM8 knockdown mice testified the significant role of MCM8 in metastasis in vivo [51].

### 2.2. MCMs as Diagnostic Markers and Therapeutic Targets

Aside from the utility as cell proliferation markers, the diagnostic potentials of the MCM proteins have been the focus of much attention. Immunostaining results and clinical practice have confirmed that most of the MCM-positive cells are distributed at the surface of malignant and premalignant epithelial cells [52], which means that the cytological specimen is easily accessible and reproducible in clinic. Given that the MCM expression level varies during cancer development, expression microarray analysis is also a feasible approach for diagnosis. Therefore, MCMs are promising biomarkers in cancer screening and diagnosis, both practically and theoretically. Diagnostic receiver operating characteristic analysis indicated that MCM2-7 proteins could serve as sensitive diagnostic markers in HCC [53]. The diagnostic values of the individual MCM subunits were identified in various cancers including MCM2 in gastric cardiac cancer and salivary gland tumor, MCM5 in esophageal cancer, MCM7 in meningiomas, and MCM10 in breast cancer [34, 46, 54–56].

As mentioned above, MCMs are a crucial replication factor acting as both a helicase and a licensing component. Moreover, MCMs may mediate DNA damage repair. Knockdown of MCM genes was reported to significantly inhibit cell proliferation [50, 57, 58]. Based on the biological and functional significance of MCMs, many MCM-targeted drugs have been developed. Anticancer therapies using the CMG complex as a target were recently reviewed by Seo and Kang [59]. Concerning the inhibition of MCM functions, two major strategies have been explored. The first strategy is to target MCM genes at transcriptional levels. It was shown that Trichostatin A, an HDAC inhibitor, and Lovastatin, an HMG-CoA reductase inhibitor, could downregulate MCM2 through activation of the JNK signaling pathway [60–62]. Also, Widdrol, an odorant compound, can activate DNA damage checkpoint through the signaling Chk2-p53-Cdc25A-p21-MCM4 pathway in HT29 cells [63]. Besides, Genistein, BETi, and Breviscapine were reported to, respectively, downregulate MCM2, MCM5, and MCM7 [61, 64]. The second strategy is to inhibit the helicase activity of MCMs. Ciprofloxacin, a fluoroquinolone, was reported to have an inhibitory effect on MCM2-7 helicase. Moreover, Heliquinomycin, an antibiotic compound, could inhibit both MCM2-7 and MCM4/6/7 trimeric helicase. It could effectively suppress cancer cell growth in lung adenocarcinoma, lung large cell carcinoma, and bladder cancer cells [65].

## 3. Prognostic Roles of MCMs

Many studies have investigated the correlation between overexpressed MCMs and adverse prognosis (Table 2). Immunostaining results of MCMs are described to be potential indicators of clinical outcomes in laryngeal squamous cell carcinoma [38], small lung adenocarcinomas [66], urothelial bladder carcinomas [67], and OSCC [68]. Gene Expression Omnibus (GEO), The Cancer Genome Atlas (TCGA), and other online cancer microarray databases combined with the Kaplan-Meier Plotter have been used to identify the unfavorable roles of highly expressed MCM10 in breast cancer [56] and hepatocellular carcinoma [53, 69]. Univariate and multivariate survival analyses with Cox's regression model present evidence for independent prognostic roles of MCM2 in LUSC prognosis [26], MCM6 in glioma [33], MCM5 in lung squamous cell carcinoma [70], and MCM8 in pancreatic cancer [31].

High expression of MCMs is reported to be negatively correlated with overall survival in many studies. However, lower expression of MCM8 is reported to be related to higher relapse rates and lower disease-free survival in Hodgkin's lymphoma [71]. In an experiment, MCM3-deficient mice were prone to hematological neoplasia [72]. Moreover, upregulated MCM2 might predict better outcome in ER-positive breast cancer [73]. The underlying mechanisms of lower and higher expression levels of MCMs in cancer prognosis could be very different.

Contrary to continuously active DNA replication caused by high-level MCMs, downregulation or defect of MCMs might induce replicative stress response (RS), which could be defined as the slowing or stalling of replication fork progression or DNA synthesis [74]. In normal cell growth, MCM2-7 is loaded at chromatin in a higher number than ORCs in the S phase, the excess MCM2-7 license DNA replication at dormant origins as a rescue strategy. When the concentration of MCM proteins decreases, the frequency of initiation at primary origins is reduced, resulting in replication fork stalling. Although initiation might be available at dormant origins, DNA damage would still accumulate because of fork progression obstacle, high frequency of chromosome recombination between nearby stalling forks, deletion of areas with tumor suppressor genes [36], and other genomic structural abnormalities [75]. The damage might lead to replication fork collapse and disrupted genomic integrity, which might cause malignant transformation in cells.

Although links between MCM expression levels and poor cancer prognosis are revealed by many studies, conclusions about the corresponding relationships remain controversial. Furthermore, some studies report that MCM2 has no independent relevance for cancer-specific survival [50, 76–78]. The most recent meta-analysis was based on 31 studies, including 7653 cancer patients. The relationship between MCMs expression and overall survival (OS) was evaluated in various cancer patients by pooled hazard ratios (HRs) and risk ratios (RRs) with 95% confidence intervals (CIs). It is shown that high expressions of MCM5 and MCM7 were significantly associated with short OS for pooled HR (HR = 1.04, 95% CI = 1.01-1.08, *P* = 0.020; HR = 1.78, 95% CI = 1.04-3.02, *P* = 0.035, respectively) and increased MCM2 and MCM7 expressions were significantly correlated with poor OS (RR = 2.30, 95% CI = 1.14-4.63, *P* = 0.019; RR = 3.52, 95% CI = 2.01-6.18, *P* < 0.001, respectively). The researchers concluded that high expression of MCM2, MCM5, and MCM7 might be prognostic indicators for poor outcomes in cancers [79].

Moreover, MCMs exhibit superiority as survival predictors. MCM7 is significantly associated with recurrence-free survival (*P* = 0.005) and obtains higher sensitivity in the ROC curve in meningioma than traditional marker MIB-1 [34]. MCM6 and MCM2 were revealed to be more accurate and reliable markers in mantle cell lymphoma [80] and esophageal cancer [81].

## 4. Molecular Mechanism of MCMs in Cancer Prognosis

### 4.1. Genomic Instability

Cancer cells are produced by accumulated mutations in oncogenes and tumor suppressor genes. Genomic variations including mutation, extensive chromosome rearrangement, deletion, and excess amplification of genes are crucial in cancer prognosis. Genomic instability resulting from MCM variations has been elucidated to have an association with cancer prognosis in many studies.

Point mutations in MCM4 (F345I, G364R, and G486D) have been reported to cause dysfunction of MCM2-7 complex, unreplicated DNA in the S phase, and segregation of structurally altered genome to daughter cells, resulting in disrupted DNA replication in daughter cells, malignancy, and relapse of carcinomas. F345I mutation weakens the interaction with MCM6 [90]. G364R mutation was detected in skin cancer cells, and G486D mutation was detected in endometrial cancer cells [1].

Copy number variations (CNVs) of MCMs contribute to genomic instability and cancer progression. The gain of MCMs gene copies might explain the overexpression of MCMs in the cancer genomes. The copy number of MCM8 reached 16 copies per genome in some samples of breast and colon cancer; the MCM8 gene amplification was also detected in epithelial-mesenchymal transition, recruitment of a more substantial proportion of cells into proliferation cycle and gain of aggressive features [51]. At the same time, a deletion event can also disrupt genomic stability through induced replicative stress response as illustrated before. It is reported that cancer progression is greatly accelerated at a critical minimum threshold of between about 35% and 50% reduction in MCM concentration [91].

In addition to point mutations and copy number alteration, MCM polymorphisms or SNPs are associated with poor prognosis as well. In the intronic region of MCM7, rs999885 controlled the expression level of miRNA (miR-106b-25) to decrease the death risk in intermediate or advanced HCC ([92]), while patients with homozygote genotype (CC) of rs1534309 showed a higher survival rate in acute myeloid leukemia than the patients with other genotypes (CG and GG) [93].

### 4.2. Molecular Interactions

#### 4.2.1. Cross-Talk in MCM Family

In an MCM2-7 complex, the six subunits interact in a defined order and are closely related to adjacent subunits. The interrelation in the MCM family is also discovered in the prognosis of various cancers. As indicators for HCC prognosis prediction, MCM2-7, MCM8, and MCM10 are significantly correlated with each other [86]. Survival analysis and joint effect analysis demonstrated that the combination of MCM2 and MCM6 could serve as HCC prognostic predictors [53]. A combination of two or more upregulated MCMs indicates a shorter disease-free survival time in RCC [27]. Based on public databases, strong positive coexpression was observed among MCM2-7 genes [94]. In triple-negative breast cancer, protein-protein interaction between Mcm2 and Mcm4 was verified with proximity ligation assay [95].

A study including seven independent breast cancer patient cohorts, each with more than 150 patients, was conducted by Kwok et al. [96] to investigate the synergistic effects among MCM family in cancer prognosis. The results showed that when considered individually, high-level MCM4 overexpression was only weakly associated with shorter survival in the combined breast cancer patient cohort (*n* = 1441, hazard ratio = 1.31, 95% confidence interval = 1.11-1.55, *P* = 0.001). On the other side, when the researchers studied all six subunits of MCM2-7 complex, overexpression of all MCMs was found strongly associated with shorter survival in the same cohort (*n* = 1441, hazard ratio = 1.75; 95% confidence interval = 1.31-2.34; *P* < 0.001). Therefore, the MCM proteins are suggested to cooperate and function as a complex in cancer prognosis. High-level expression of the whole MCM complex might have higher robustness in indicating adverse clinical outcomes of cancer.

#### 4.2.2. Interactions with Other Molecules

*Geminin* Geminin is a protector of genomic integrity that inhibits the reinitiation of DNA replication in the same cell cycle. Geminin binds to MCM loader Cdt1 protein and thus blocks the reloading of MCM2-7. The balance of geminin to Cdt1 and its impact on MCM loading may contribute to oncogenesis [97]. It is reported in breast carcinoma that geminin is only expressed from S to M phase [98]. In this case, geminin can identify the actively proliferating proportion of cells that have entered the S phase yet not exited mitosis. Therefore, geminin can be used to indicate the rate of cell-cycle progression and screen cells with malignant transformation tendency, and serve as a potential prognostic marker. Immunohistochemical analysis has demonstrated that geminins are useful prognostic predictors in colorectal cancer [99].

Both MCM-positive and geminin-positive phenotypes could be observed in cancer tissues, and as the differentiation degree elevated, the decoupling level of MCM and geminin increased correspondingly [100]. Tumor progression-free survival and multivariate analysis identified the MCM7-geminin status as an independent prognostic factor in laryngeal SCC rather than MCM7 and geminin covariates separately [88]. As an indicator of the cell cycle state, the geminin/MCM fraction was suggested to provide a better prognostic value, because the rate of tumor cell proliferation and tumor growth could be represented by the ratio [52].

*GINS* GINS (go-ichi-ni-san) is a tetramer comprising four small subunits (Sld5 (Cdc105), Psf1 (Cdc101), Psf2 (Cdc102), and Psf3 (Cdc103)—“go, ichi, ni, san” means “5, 1, 2, 3” in Japanese). GINS is required for the MCMs' regular functioning. GINS interacts with MCM2-7 and Cdc45 to form the CMG complex [5]. In the CMG complex, GINS must be stably associated with MCM2-7 to facilitate the interaction between the helicase domain of MCM2-7 and key regulatory proteins in replisome progression complexes (RPCs), thus promoting the establishment of DNA replication forks. RPC proteins include the essential initiation and elongation factors, the checkpoint mediator Mrc1, the histone chaperone FACT (facilitates chromatin transcription), and Ctf4, which helps to establish sister chromatid cohesion. Some of these RPC proteins might also interact with MCM10 during the progression of DNA replication forks with the help of GINS [101]. Survival analysis showed that either the GINS subunit alone or the GINS complex as a whole could serve as a potential prognostic biomarker for HCC [69]. The coexpression and synergistic effect of GINS and MCMs in cancer merit further exploration.

*Other Molecules* In addition to geminin and GINS, many other molecules may work in combination with MCMs in prognostic prediction. IDH1 mutation, a classical biomarker in glioma, was reported to combinate MCM6 overexpressions to improve the prediction of the prognosis in glioblastomas [33]. MCM4 and MCM7 expressions were significantly associated with Ki-67, Bmi1, and cyclin E expression in esophageal carcinoma prognosis [47]. The proteins functioning in DNA replication licensing, including Cdc45, CDC6, Cdt1 CDK, and DDK, might have combinational effects with MCMs in cancer prognosis. Further research is required to explore the interaction model of MCMs and other molecules in prognosis.

#### 4.2.3. Effects of MCMs in Cellular Signaling Pathways

As mentioned above, the available studies indicate that MCMs function in tumorigenesis and cancer prognosis through DNA replication initiation and replicative stress response. In recent studies, functional enrichment analysis has been used to explore the roles of MCMs in other cellular biological processes and pathways. A single-gene GSEA (gene set enrichment analysis) investigation for MCM4 implied that MCM4 might participate in multiple cellular processes including DNA replication, cell cycle, nuclear division, tumor protein p53, Notch signaling pathways, and pathways in cancer [94]. MCM2-7 were all reported to be significantly enriched in DNA replication and cell cycle [53].

**Figure 2:**
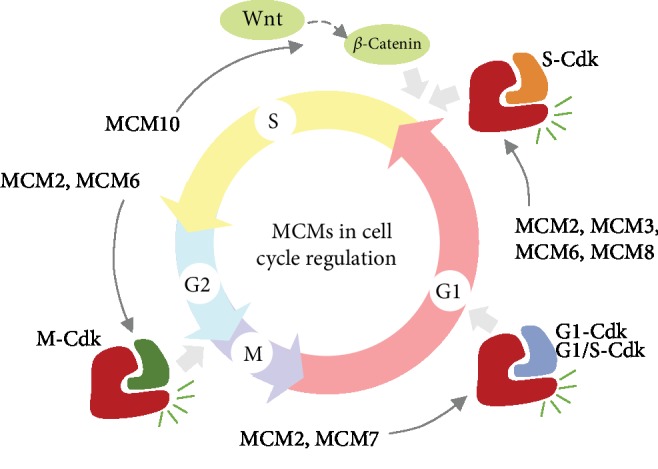
Effects of MCMs in pathways of cell cycle progression. Cyclins and Cdk partners of each cyclin-Cdk complex are listed as follows: G1-Cdk: cyclinD, Cdk4, and Cdk6; G1/S-Cdk: cyclinE and Cdk2; S-Cdk: cyclinA, Cdk1, and Cdk2; M-Cdk: cyclinB and Cdk1.

Many studies have provided cytological evidence for bioinformatics analysis that MCMs are involved in the progression of cell cycle pathways (Figure 2). Knockdown of MCM2 reduced Rb, cyclinD1, cyclinA, and CDK4 expression and increased p53 and p21 expression, suggesting that by inducting the Cdk inhibitor p21cip14 and cyclin D/CDK4, downregulated MCM2 triggered the arrest of G1/S or G2/M [50, 62]. Knockdown of MCM3 caused G1 arrest as evidenced by reduced expression of cyclinA [50]. Knockdown of MCM6 was related to a delay in S/G2-phase progression with downregulation of CDK2, CDK4, CyclinA, CyclinB1, CyclinD1, and CyclinE in HCC [86]. Silencing of MCM7 reduced cyclinD1, cyclinE2, and CDK2 [58], which triggered events in the G1 or G1/S phase. Another experiment indicated that MCM8 bound to cyclin D1 to active Rb protein phosphorylation by Cdk4, and the binding allowed the entry into the S phase [51]. Besides, results of western blot analysis showed that *β*-catenin and cyclin D1 were downregulated in MCM10 knockdown cells, suggesting that downregulated MCM10 suppressed metastasis in breast cancer via the Wnt/*β*-catenin pathway [56]. The Wnt/*β*-catenin pathway is involved in cell proliferation and differentiation. Wnt signaling regulates the transition from G1 to S phase in mitosis; excessive Wnt signaling mediated *β*-catenin elicit activation of oncogenes like cyclinD1 [102]. Therefore, MCM10 may promote the G1/S transition through activation of the Wnt/*β*-catenin pathway.

MCMs are also involved in apoptosis pathways and inhibit some apoptotic proteins. In triple-negative breast cancer, MCM2-7 complex was reported to participate in a high mutant p53-chromatin-associated pathway [95]. In this research, a protein-protein interaction between overexpressed mutant p53 and MCM2/4 was directly detected. Moreover, MCM2-7 was required for synergistic mutant p53-dependent induction of apoptosis by the combination of the poly ADP-ribose polymerase inhibitor talazoparib and the alkylating agent temozolomide, indicating a mutant p53-poly-ADP-ribose polymerase minichromosome maintenance functional axis. The antiapoptotic protein Bcl-2 was also reported to have an association with MCM expression, but it remained controversial whether the expression levels of Bcl-2 and MCMs were positively related [103, 104].

Likewise, MCMs may participate in cellular metabolism pathways. AKT is a serine/threonine kinase involved in the regulation of cell metabolism, proliferation, and survival. Silencing of MCM7 inhibited the phosphorylation of AKT1 and mTOR in both esophageal cancer cell lines, suggesting that MCM7 might promote cancer development and poor survival outcomes via activating the AKT1/mTOR signaling pathway [58]. Implicated genes and pathways in lymphoblastic lymphoma of MCM2-deficient mice include Pten, Tcfe2a, Mbd3, Setd1b, and Notch signaling pathway [36]. Broader bioinformatic, cytological, and clinical studies of MCMs in cellular signaling pathways should be performed in various carcinomas to further understand the interaction networks of MCMs.

### 4.3. Protein Modification

MCMs are regulated in cell cycle through protein modification. Improper modifications might be mechanisms for cancer development and adverse prognosis.

*Phosphorylation* MCMs undergo phosphorylation in three major cellular processes: DNA replication initiation, replicative stress response, and cell cycle checkpoint response. They are phosphorylated by Cdc7, Cdk, and ATM/ATR, respectively, in these processes. Phosphorylation activates MCM proteins and promotes the progression of the processes. Dysfunctional phosphorylation of MCMs is suggested to be associated with oncogenesis and cancer development [105].

*Sumoylation* Yeast MCMs undergo sumoylation that negatively regulates DNA replication initiation. MCM sumoylation levels reach the highest in G1. Subsequently, MCM sumoylation levels decrease as the phosphorylation levels increase in the S phase. MCM sumoylation suppresses MCM phosphorylation and activation through the recruitment of phosphatase. The balance between sumoylation and phosphorylation is vital for accurate and flexible control of replication initiation [106]. Restrained ubiquitylation in eukaryotic cells may expedite the uncontrolled proliferation of cancer cells.

## 5. MCMs in Liver Cancer

Liver cancer is the fourth deadliest cancer worldwide, while lack of prognostic indicators and poor overall survival remains problematic. MCMs have been suspected to be involved in hepatocarcinogenesis and hepatocellular carcinoma (HCC) prognosis in previous studies. Quantitative polymerase chain reaction (qPCR) was utilized in 105 samples (normal livers (*n* = 15), cirrhotic livers (*n* = 40), and HCC (*n* = 50)) and identified that MCM2-7, MCM8, and MCM10 were significantly upregulated in HCC [86]. The result is consistent with the coexpression of these genes in HCC tissue in the GSE14520 and TCGA cohort [53].

Survival analysis in GSE14520 and TCGA cohort showed that among the upregulated genes, only MCM2 and MCM6 were significantly associated with both HCC overall survival and recurrence-free survival in both cohorts [53]. It is further validated by PCR and parallel immunostaining that MCM6 has superiority in diagnosis and prognosis over MCM2 and other MCMs in HCC [86, 87]. Moreover, high-level expression of MCM6 is correlated to vascular invasion, lymph node metastasis, and tumor stage progression in HCC development, especially in AFP negative and small HCC patients [87].

Multiple evidences support the fact that MCM6 is a promising independent biomarker in HCC. However, few studies have taken a look inside the mechanism of MCM6 in HCC development and prognosis. A previous study indicated that MCM6 triggered the S/G2 phase transition [86]. Besides, GINS subunits, which interact directly with MCMs at pre-RC during initiation, may also be prognostic indicators in HCC [69]. Further research should be conducted to investigate the roles of MCMs in HCC.

## 6. Conclusion and Perspectives

MCMs are of great importance in double-strand DNA unwinding, DNA replication control, and DNA damage repair. Upregulated MCM genes were detected in various cancers, and cell proliferation was significantly inhibited in multiple MCM knockdown cancer cell lines. Therefore, MCMs are regarded as promising diagnostic and prognostic markers, as well as potential targets for anticancer therapy. In this review, we have discussed the functional and prognostic significance of MCMs by considering evidence from original studies and meta-analytical researches in multiple types of cancer cells. Furthermore, we have amplified the MCM-related molecular mechanisms and their relevance to tumorigenesis and adverse prognosis.

Overexpressed MCM proteins were identified as proliferation markers and diagnostic indicators in many cancers. Based on the biological functions, several MCM target drugs were designed to either decrease transcriptional levels of MCM genes or inhibit the helicase activity of MCM2-7. In the majority of types of cancers, high expression of MCMs has emerged as powerful prognostic indicators. Specifically, MCM6 is implied as the most valuable biomarker in HCC among MCM family. It is expected to be examined by larger-scale research, and the mechanisms remain to be explicated. Even though MCMs are recognized as promising prognostic factors, due to types of cancer, scoring variability, and analytical validity between research groups, precise cut-off values in different cancers are still needed to be determined in clinical settings.

MCMs may function in adverse prognosis through dysregulated initiation and uncontrolled replication licensing. They may also interfere with cancer recovery via genomic instability, molecular interactions, and protein modification. In addition to chromosome rearrangement in RS, point mutations, copy number alteration, and SNPs might all be sources of genomic instability. Interactions within the MCM family, interactions with other factors, and participation in cellular signaling pathways may all contribute to the prognostic outcomes. Current evidence shows that silence of some MCM genes is related to cell cycle arrest in different points (as discussed in review). However, behaviors and significance of the MCM family in different periods of interphase have not been systematically investigated. Comprehensive understanding of MCMs in cell cycle pathways may provide innovative means for cell cycle control and new therapeutic strategies. Besides, since phosphorylation is crucial for MCM activity regulation and dysfunctional phosphorylation of MCMs appears to correlate with the occurrence and development of cancers, Fei and Xu [105] have proposed that protein kinase inhibitors can be used therapeutically to target MCM phosphorylation in cancer. Considering the cross-talk between phosphorylation and sumoylation, as well as the proliferation-promoting effect of restrained ubiquitylation, manipulation of sumoylation and ubiquitylation may be a potential method for MCM-targeted cancer therapy.

## Figures and Tables

**Table 1:** Summary of biological functions of the MCM subunits.

Subunit	Biological and functional significance	References
MCM2	Chaperones histones H3-H4. Dephosphorylated by PTEN to restrict replication fork progression. Phosphorylated by DDK at NTD terminus to activate pre-RC. Interacts with Cdt1 at NTD to stabilize the coil structure. Interacts with Cdc45 in the CMG complex at NTD. Forms MCM2-5 gate for the MCM2-7 complex loading.	[9] [10] [11] [4] [12] [13]

MCM3	Interacts with GINS component Psf2 in the CMG complex. Interacts with and stimulates the ATPase activity of ORC·Cdc6 at CTD.	[12] [14]

MCM4	Phosphorylated by DDK at NTD to activate pre-RC. Interacts with Cdt1 at NTD to stabilize the coil structure. Involved in translocation along single-stranded DNA in the MCM2-7 complex.	[11] [4] [1]

MCM5	Interacts with GINS component Psf3 in the CMG complex. Interacts with Cdc45 in the CMG complex at NTD. Forms MCM2-5 gate for the MCM2-7 complex loading.	[12] [15] [13]

MCM6	Phosphorylated by DDK at NTD terminus to activate pre-RC. Interacts with Cdt1 at NTD to stabilize the coil structure. Involved in translocation along single-stranded DNA in the MCM2-7 complex.	[11] [4] [1] [1]

MCM7	Involved in translocation along single-stranded DNA in the MCM2-7. Polyubiquitylated during replication termination.	[1] [16]

MCM8	Functions as a helicase during replication elongation. Stabilizes MCM9 and forms MCM8-9 complex to facilitate homologous recombination.	[17] [18]

MCM9	Forms MCM8-9 complex to facilitate homologous recombination. Functions in germ-line stem cells and tumor suppression.	[18] [19]

MCM10	Functions as one of the initiation factors to activate the helicase activity of MCM2-7 complex. Stabilizes Cdc45 and GINS association with Mcm2-7 and stimulates replication elongation. Coordinates DNA helicase and polymerization activities during lagging strand synthesis.	[20] [21] [22]

**Table 2:** Prognostic roles of the MCM proteins in different types of cancer.

Subunit	Tumor types	References
Negative correlation between MCM expression level and prognostic outcomes		
MCM2	Lung squamous cell carcinoma, glioma, muscle-invasive urothelial bladder carcinomas, ovarian adenocarcinomas, prostate cancer, diffuse large B-cell lymphoma, salivary gland tumor, hepatocellular carcinoma, gastric cardiac cancer	[26], [82], [67], [37], [83], [84], [46], [53], [54]
MCM3	Glioma, oral squamous cell carcinoma, cutaneous T-cell lymphomas	[82], [44], [85]
MCM4	Laryngeal squamous cell carcinoma	[38]
MCM5	Lung squamous cell carcinoma, muscle-invasive urothelial bladder carcinomas, ovarian adenocarcinomas	([37, 67, 70]
MCM6	Glioma, hepatocellular carcinoma	[33], ([86]; [87])
MCM7	Colorectal cancer, glioma, oral squamous cell carcinoma, laryngeal squamous cell carcinoma, diffuse-type gastric adenocarcinoma, papillary urothelial neoplasia	[77], [82], [68], [88], [43], [39]
MCM8	Pancreatic cancer	[31]
MCM10	Breast cancer, hepatocellular carcinoma, urothelial carcinoma	[56], ([53]; [69]), [57]
Positive correlation between MCM expression level and prognostic outcomes		
MCM2	ER-positive breast cancer, colorectal cancer	[73], [89]
MCM6	Hodgkin's lymphoma	[71]
MCM7	Small lung adenocarcinomas	[66]
Not qualified to be an independent prognostic marker		
MCM2	Colorectal cancer, squamous cell carcinoma of the penis, non-small-cell lung cancer, medulloblastoma	[77], [76], [78], [50]
